# Self‐perception of overweight and obesity: A review of mental and physical health outcomes

**DOI:** 10.1002/osp4.424

**Published:** 2020-06-08

**Authors:** Eric Robinson, Ashleigh Haynes, Angelina Sutin, Michael Daly

**Affiliations:** ^1^ Psychological Sciences University of Liverpool Liverpool UK; ^2^ Centre for Behavioural Research in Cancer Cancer Council Victoria Melbourne VIC Australia; ^3^ College of Medicine Florida State University Tallahassee Florida USA; ^4^ UCD Geary Institute for Public Policy University College Dublin Dublin Ireland; ^5^ Behavioural Science Centre University of Stirling Stirling UK

**Keywords:** obesity, self‐regulation, stigma, weight perception

## Abstract

The obesity crisis is one of the largest public health challenges of the 21st century. Population‐level adiposity has increased dramatically in recent times, and people not recognizing that they have overweight or obesity is now common. It has been widely assumed that not recognizing oneself as having overweight is detrimental to weight management and long‐term health. Here, diverse research is reviewed that converges on the counterintuitive conclusion that not recognizing oneself as having overweight is actually associated with more favourable physical and mental health outcomes than recognizing oneself as having overweight. Drawing on existing models in social psychology and weight stigma research, an explanatory model of the health effects of self‐perception of overweight is outlined. This model proposes that self‐perception of overweight triggers social rejection concerns and the internalization of weight stigma, which in turn induce psychological distress and negatively impact health‐promoting lifestyle behaviours. How self‐perception of overweight may in part explain progression from overweight to obesity, and the public health implications of self‐perception of overweight and obesity are also discussed.

## 1 INTRODUCTION

In the last 30 years, most of the developed world has witnessed mass population‐level weight gain.^1^ For example, the majority of adults in the United States and England are now considered medically to have overweight (a body mass index [BMI] of 25–29.9 kg/m^2^) or obesity (a BMI of 30 kg/m^2^ and above).^2^, ^3^ Living with obesity is thought to reduce life expectancy by as much as 10 years by negatively impacting the body's vital systems for health, especially cardiovascular and metabolic health.^4^, ^5^ Although the health consequences of heavier body weight are more pronounced among people classed as having obesity, overweight is also associated with an increased risk of a range of health conditions, including hypertension and diabetes.^6^, ^7^, ^8^ The obesity‐related disease burden is now sizeable and increases in population body weight has been identified as one reason why life expectancy is no longer rising steeply in developed countries.^9^ To date, the obesity‐related disease burden has been explained largely by an organic model of disease, whereby adiposity places several of the body's vital systems under physiological strain, and this process causes ill health.^10^, ^11^ This article provides an overview of the health implications of people identifying versus not identifying that they have overweight.

## 2 KNOWING VERSUS NOT KNOWING

A consequence of population‐wide weight gain and the increased prevalence of obesity is that heavier body weights have become more “normal.” Based on population statistics, more than one in every three adults in the United States are now medically considered to have obesity, and therefore, people are more frequently exposed to larger body sizes.^2^ Evaluations about a person's weight are made relative to the range of bodies perceived as being normal in size.^12^, ^13^ It has been proposed that there has been a widespread “normalization” of heavier body weights and thisresulted in a number of people whose objective BMI is in the “overweight” weight range not recognizing that they have overweight.^14^ In support of this, a sizeable proportion of people in developed countries (approximately one quarter to half depending on study) who are medically defined as having overweight do not identify themselves as such and instead believe their weight is “about right.”^15^, ^16^ For example, recent estimates in England suggest that more than one in three men and one in five women with overweight or obesity underestimate their weight status.^17^ In support of the normalization hypothesis, studies have shown that personal underestimation of weight status has increased as obesity has become more common.^17^, ^18^ Likewise, not identifying oneself as having overweight has been shown to be more common among people with more social contacts who are of heavier body weight.^19^, ^20^

There is an extensive body of research on body image and heavier body weight.^21^ Of most relevance to perceptions of weight status, it has been shown that large numbers of people with overweight or obesity do not accurately identify their weight status and this has been described as a major public health concern and barrier to successful weight management.^18^, ^22^ This line of reasoning is in fitting with the assumption both implicit and explicit in many theoretical models of health behaviour (e.g., health belief model and transtheoretical model of change): that awareness of a medical condition is required for effective management or treatment.^17^ Based on this reasoning, underestimation of weight status may result in individuals failing to address their diet and/or physical activity, which facilitates the maintenance of an unhealthy body weight or further weight gain, as opposed to weight loss. This widely held belief has informed obesity intervention efforts. For example, in school and health care settings and public health websites in the United States and United Kingdom, it is common to provide feedback on the objective weight status of children and adults,^23^, ^24^, ^25^ under the assumption that correcting misperceptions about weight status should motivate better weight management.

The present article argues that the widely held belief that a person not recognizing that they have overweight will be detrimental to their long‐term health is wrong. Instead, evidence is reviewed that suggests that not identifying oneself as having overweight is actually associated with better long‐term weight management and health outcomes than accurately perceiving oneself as having overweight. As this is a theoretical review of a range of study types, we did not use systematic literature review methodology (e.g., database searches and formal article eligibility assessments) but drew upon available systematic reviews and meta‐analyses where available.

## 3 THE STIGMA OF HEAVIER BODY WEIGHT

There is widespread stigmatization of heavier body weight in the Western world. Heavier body weight is viewed by many as signalling personal failure, and those with obesity are portrayed as lacking self‐control and intellect by popular media^26^. These negative stereotypes attributed to heavier body weight appear to be widely endorsed, as a number of studies show both negative implicit and explicit attitudes towards people of heavier body weight among both the general public and health care professions.^27^, ^28^ Stigma towards heavier body weight is now recognized to be ubiquitous.^29^ The extent to which obesity is stigmatized is highlighted by recent evidence that indicates that people living with obesity are rated as less human and evolved because of their heavier body weight.^30^ A consequence of the widespread stigmatization of adiposity is that being overweight or obese is highly undesirable. For example, in one study, 30% of participants reported that they would rather be divorced than have obesity, and 25% reported that they would rather be unable to have children than have obesity.^31^ Social identity theory proposes that a person's sense of worth is in part derived from the social groups or categories that they belong to.^32^ Directly informed by social identity threat theory,^32^ it is proposed that the widespread and socially acceptable stigmatization of heavier body weight has negative consequences for people who identify as being overweight. People who identify their body weight as being “overweight” recognize that they possess a personal characteristic that is stigmatized by others, and this recognition may negatively affect physical and mental health.

## 4 IS IT BETTER TO KNOW OR NOT KNOW?

As discussed, underestimation of weight status among people living with overweight or obesity has been argued to facilitate maintenance of an unhealthy body weight or even promote further weight gain because not identifying as overweight may result in little motivation to manage one's body weight. Indeed, people who have been diagnosed as having obesity or who accurately identify that they have overweight do appear more motivated to manage their weight, as self‐identification of overweight is associated with greater self‐reported weight loss intentions and/or attempts to lose weight.^15^, ^33^ Until recently, whether or not these intentions resulted in improved weight management had not been examined. A series of three studies including US and UK participants found that as compared with people who do not identify that they have overweight, self‐identification of overweight is predictive of increased weight gain over follow‐ups of 5–20 years,^34^ irrespective of whether self‐identification was accurate (people with overweight or obesity) or inaccurate (people with normal weight). The association between self‐identification of overweight (measured at baseline) and worse weight management over time was mediated in part by a baseline measurement of stress induced eating.^34^ Identifying oneself as having overweight was associated with an increased likelihood of overeating to cope with stress, and this maladaptive coping strategy predicted increased weight gain. This finding supports the hypothesis that the stigma and stress of self‐identifying as being overweight may compromise health.

A recent systematic review confirmed the replicability of the association between self‐perception of overweight and increased weight gain across multiple studies and also showed that identification of overweight places a person at an increased risk of a range of behaviours that impede weight management, including disordered eating patterns.^35^ Recent findings also suggest that there may be an intergenerational effect of perceived weight status on weight gain. Children identified by their mothers as having overweight tend to gain more weight across childhood as compared with similar children whose mothers do not recognize they have overweight.^36^, ^37^ Consistent with findings linking self‐identification of overweight to weight gain, the effect of parental perception on child weight gain has been shown to be in part explained by parental perception increasing the likelihood that their child identifies themselves as having overweight.^37^

Moving beyond weight management, an accumulation of evidence suggests that self‐identifying as overweight places individuals at increased risk of adverse mental health outcomes. A number of cross‐sectional studies show that people who identify that they have overweight are more likely to experience depressive symptoms, anxiety, and poorer overall psychological well‐being.^38^, ^39^ The associations are not only cross‐sectional. A recent systematic review and meta‐analysis showed that self‐identification of overweight, as opposed to not identifying oneself as having overweight, is a prospective risk factor for the development of depression and attempted suicide.^40^ Moreover, the negative associations between self‐identification of overweight and both increased weight gain and mental health problems are observed consistently among men and women, adolescents and adults, and irrespective of objective body weight.^35^, ^40^

The findings are consistent, but the majority of the research literature linking self‐identification of overweight with weight‐related behaviour and mental health outcomes relies on observational designs. Although research indicates that people who identify as having overweight gain more weight and experience worse mental health in future than people who do not, these studies cannot provide causal evidence for the potential damaging effects of self‐identification of overweight. There is, however, a small amount of experimental research that has examined weight and mental health‐related consequences of the psychosocial experience of identifying and feeling overweight. Two studies found that informing people that they are overweight resulted in increased negative affect and lower self‐esteem.^41^, ^42^ Further, a series of studies has examined the consequences of temporarily causing participants to “feel” overweight. In these studies, participants were randomized to a control condition or a condition in which they were asked to wear a body prosthetic under their clothing that results in them appearing as a person with obesity and then walking in public. The obesity prosthetic resulted in participants reporting feeling overweight and mimicked some of the psychosocial experience of identifying as a person with overweight. Participants, for example, reported feeling conscious of their appearance and feared being stigmatized by others because of their apparent body size.^43^, ^44^, ^45^ Studies using this paradigm have shown that causing participants to feel overweight increased negative affect, impaired self‐control, and, in four out of five studies, it increased overeating when participants were provided with high‐calorie snack food.

Examining longer‐term consequences of self‐identification of overweight using experimental designs has been rare, but using a quasi‐experimental regression discontinuity design^46^ a study found that BMI report cards informing medically defined overweight New York City public school students that they are overweight resulted in inadvertently promoting a small level of weight gain among girls, with no effect in boys. That is, girls who fell just over the threshold for overweight (and thus labelled overweight in the BMI report card) gained more weight than girls who fell just under the threshold for overweight (and thus labelled normal weight in the BMI report card). Although there was not a similar effect for boys, boys who were labelled as overweight did not lose weight over time, as would be expected if knowledge of overweight promoted healthier weight management. As such, informing individuals of their weight status at best has no effect on long‐term weight management and promotes greater weight gain at worse. Similarly, a recent evaluation of the Korean National Health Screening Program showed that receiving information on being at high obesity risk (based on the presence of abdominal obesity and BMI > 25 kg/m^2^) was unrelated to changes in waist circumference or physical activity or the level of medical treatment individuals received. In contrast, the regression discontinuity design revealed that those just above the threshold for receiving feedback that they were at medium risk of obesity (no abdominal obesity, BMI > 23 kg/m^2^) went on to experience greater increases in waist circumference than those just below the threshold who received a low risk classification.^47^

There is a lack of experimental data examining the consequences of self‐identification of overweight, and this is critical to inferring causality. However, the experimental and quasi‐experimental data reported on to date largely corroborate the findings from observational studies: self‐identification of overweight is not associated with better weight‐management but rather poorer mental health and worse weight‐related outcomes over time.

## 5 HOW COULD KNOWING HURT?

**FIGURE 1:**
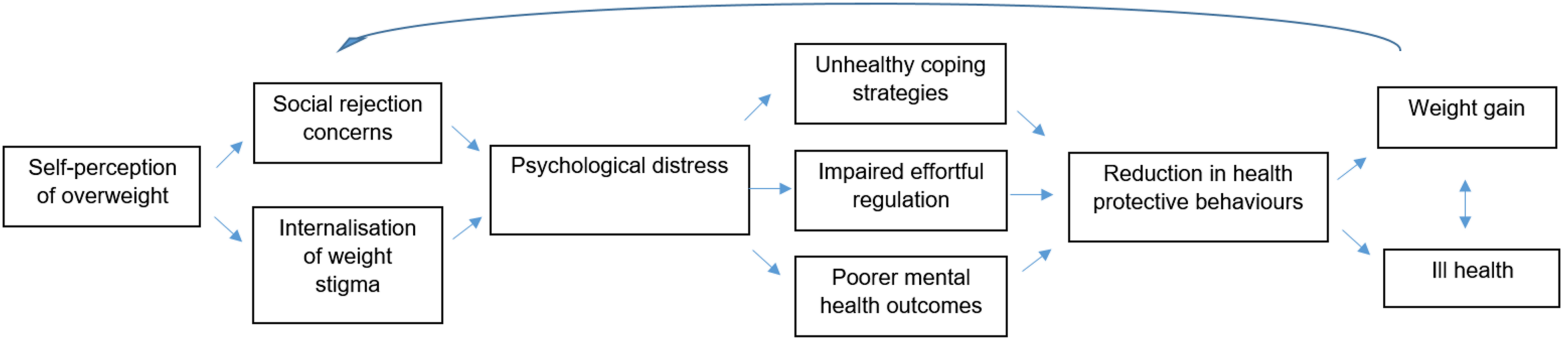
Proposed model of self‐identification of overweight and health outcomes

Figure 1 outlines a pathway model to explain why people who self‐identify that they have overweight are more likely to go on to experience worse mental and physical health than those who do not. This model directly draws on previous theoretical frameworks designed to explain how the stigma of heavier body weight may cause ill health, including the Cyclic Obesity/Weight‐Based Stigma model (COBWEBS) and the Weight‐Based Social Identity Threat model.^48^, ^49^

This model proposes that the widespread and socially acceptable stigma associated with heavier body weight results in people who self‐identify as having overweight being aware that they possess a personal characteristic that is socially devalued,^29^ and this model suggests that this will have two key psychological effects: fear of social rejection and internalization of stigma. Stigmatized social groups often fear rejection from others because of their devalued identity^32^ and will often internalize the negative stereotypes and beliefs associated with that devalued identity. There is evidence that people who self‐identify as having overweight internalize the stigma associated with heavier body weight (“I hate myself because of my weight”)^50^ and report fearing social rejection from others because of their body weight (“I think others will judge me negatively because of my weight”)^51^: findings consistent with the proposition that personal identification of being overweight is likely to change how people view themselves (internalized weight stigma) and how they expect others to view them (social rejection concerns). These psychological consequences may be critical to understanding why self‐identification of being overweight is associated with subsequent ill health because both factors greatly increase the likelihood of a person more frequently experiencing psychological distress, in the form of stress and negative emotion. For example, there is evidence that concerns about social rejection, including rejection based on appearance, are linked to more frequently experiencing distressing negative emotions and poorer psychological well‐being.^52^, ^53^, ^54^ Likewise, internalization of stigma is a well‐studied construct across a range of social stigmas, and it has been shown to be associated with the experience of greater psychological distress among those living with a stigmatized social identity.^55^, ^56^

There are meaningful health consequences to frequently experiencing negative emotions and psychological distress.^57^, ^58^, ^59^ There are likely to be a myriad of factors that result in the psychological distress caused by self‐identifying as having overweight leading to behaviours which impair, rather than protect, against health. First, a common coping response to negative emotion and stress is to comfort eat, which is a known risk factor for weight gain^60^ and, as discussed, has been shown to explain the link between self‐identification of overweight and weight gain.^34^ A second factor that may explain why the psychological distress associated with identifying as having overweight could impair health is because it increases weight‐loss attempts. A different approach to coping with the psychological distress of identifying oneself as having overweight is to attempt to escape the stigma of being an overweight person by losing weight. However, long‐term weight loss is difficult to achieve and some researchers have gone as far to argue that dieting often “backfires” and people who attempt to lose weight will often end up gaining more weight than if they had not attempted weight loss.^61^ Likewise, backfiring weight‐loss attempts may promote unhealthy coping strategies (see Figure 1). Moreover, the psychological resources and skills needed to achieve long‐term weight loss, such as effortful self‐control and self‐regulation, are impaired by feelings of stigma, distress, and negative emotion (see Figure 1), which in turn interfere with efforts to engage in behaviours conducive to healthy weight, for example, greater healthy eating and physical activity.^62^, ^63^

A set of prospective studies have now uncovered evidence in support of this backfiring hypothesis—identifying as having overweight is associated with an increased likelihood of attempting weight loss,^35^ which in turn forecasts weight gain rather than weight loss over time. As such, more frequent weight loss attempts partly explain why people who identify as having overweight go on to gain more weight than those who do not.^64^ A third pathway in the model explaining why the stigma of identifying as having overweight can result in behaviours that impair rather than protect against ill health is through the direct impact that stigma can have on mental health. Because of the psychological distress and shame attached to stigmatized identities, people who are part of stigmatized social groups are at an increased risk of developing a range of mental health conditions such as depression.^32^, ^65^ In a similar vein, self‐identification of overweight (as opposed to not identifying) places a person at increased risk of developing depression.^40^ Mental health conditions like depression make many health‐protective lifestyle behaviours more difficult to achieve, including regular physical activity, healthy eating, and adequate sleep.^66^, ^67^ Thus, the psychological distress of self‐identifying as having overweight is likely to negatively impact patterns of behaviour that promote, rather than protect against, ill health.

Critical to the proposed model is that these patterns of behaviour will be to the detriment of weight management, as lifestyle behaviours such as comfort eating^60^ and reduced physical activity^68^ are associated with an increased risk of further weight gain, which in turn will then negatively impact health. However, many of these behaviours are also thought to act on health directly, as risk of cardiovascular disease, cancers, and other chronic health conditions can be lowered or raised by these lifestyle behaviours independent of body weight.^69^, ^70^ Although relatively few investigations have examined whether self‐identification of having overweight is a direct risk factor for ill health, emerging evidence suggests it may be.

In one study, accounting for actual body weight, US participants who self‐identified as having overweight were more likely than participants not self‐identifying as being overweight to have an underlying physiological profile (e.g., cardiovascular, metabolic, and immune function) associated with increased risk of future ill health.^71^ Another study drawing on the same US sample showed that adolescents with overweight and obesity who self‐perceived as having overweight went on to experience raised blood pressure levels relative to those who self‐perceived as normal weight.^72^ Likewise, in a study of Korean adults, participants who self‐identified as being overweight or obese showed a worse profile for biological markers of underlying metabolic health than participants who do not identify themselves as having overweight.^47^ From these studies, it is not clear whether self‐identification of overweight acts on biological health indirectly through unhealthy lifestyle behaviours or via a more direct pathway involving a chronic biological stress response. Nonetheless, these findings raise the possibility that merely being aware that you have overweight or obesity may produce some of the negative physiological effects that have traditionally been assumed to be caused by the physical consequences of heavier body weight.

## 6 DEVELOPMENTAL CONSIDERATIONS

In line with the COBWEBS model,^49^ there may be a feedback loop between self‐identification of overweight and weight gain, and this feedback loop has developmental implications. Based on the feedback loop (see Figure 1), a novel proposition is that self‐identification of having overweight may be an important psychological process that explains why some children and adults gain weight and progress from being medically overweight (BMI 25–29.9 kg/m^2^) to developing obesity (BMI ≥ 30 kg/m^2^). A sizeable proportion of both children and adults who are medically defined as having overweight do not identify they are overweight and whether or not a person does self‐identify as having overweight will be determined by a range of factors. Although it seems likely that some people will change their weight status perception from being “normal weight” to being “overweight” during adulthood, adolescence is a key period in which self‐image is developed. Adolescents will form perceptions of their own body size in response to media exposure, how their weight compares with others in their social circle, and parental behaviour.^14^ In line with this development perspective, a parent identifying their child as being overweight at age 8 years predicts that children perceiving themselves as being overweight by age 12 years.^37^ Therefore, adolescence may be a particularly important development period in which the damaging effects of self‐identifying as overweight begin.

Having self‐identified as overweight, to progress from having overweight to obesity, a person's energy intake needs to increase and/or their energy expenditure needs to decrease for a prolonged period. As discussed, self‐identification of overweight is predictive of a range of behaviours that promote increased energy intake (e.g., increased overeating) and/or decreased energy expenditure (e.g., reduced physical activity). Thus, when a person first self‐identifies that they have overweight, this may set in motion a myriad of effects that place them on the road to further weight gain and obesity. Based on the feedback loop outline in Figure 1, weight gain would then amplify concerns over social rejection and internalization of weight stigma, which in turn would heighten psychological distress and impair effortful self‐regulation of behaviour.^49^ Self‐identification of having overweight may therefore play a role in the development of obesity. Likewise, self‐identification of overweight may also play a role in the development and maintenance of mental health problems. Several studies suggest that the well‐established tendency of heavier body weight being associated with an increased risk of depression is largely shaped by whether or not a person self‐identifies that they have overweight.^40^, ^73^ This finding is consistent with the proposition that self‐identification of overweight may serve to maintain mental and physical health difficulties experienced by people of heavier body weight.

## 7 SELF‐IDENTIFICATION OF OVERWEIGHT VERSUS OBESITY

To date, research has tended to examine the health correlates of identifying versus not identifying oneself as having overweight. There has been far less research specifically examining self‐identification of having obesity. It has been suggested that few adults with obesity recognize that they have obesity. For example, data collected in 2012 indicate that less than 10% of UK adults with obesity identified their weight status (“obese”) accurately, but the majority of participants in this study did at least recognize themselves as being overweight.^74^ Although the stigma of self‐identifying as having obesity may be even more stressful than self‐identifying as having overweight, the weight of evidence reviewed here suggests that self‐identification of overweight in itself is a likely cause of distress among people who are objectively classified as having overweight or obesity. However, because self‐perceived overweight becomes more likely at heavier body weights,^18^ the proposed model is of relevance to people with obesity, as a large proportion will self‐identify as overweight. Although untested, an intriguing possibility is that this may also contribute to why the health effects of adiposity are sometimes inconsistent among people with objective overweight (a subpopulation in which not self‐identifying as overweight is relatively common), but more reliable and pronounced among people with obesity (a subpopulation in which nearly all self‐identify as being overweight or obese).^75^

## 8 THEORETICAL CONTEXT

The proposed model explains the discussed associations between self‐identification of overweight, health behaviour, and the development of obesity, and as such it is based on existing theoretical work on the stigma of obesity. Stereotype threat theory^76^ and more specifically, weight‐based social identity theory^48^ and the Cyclic Obesity/Weight‐Based Stigma (COBWEBS) model,^49^ directly informs the emphasis on how self‐identification of overweight may impact health due to the negative effects that social rejection concerns and chronic stress have on health‐promoting behaviours. Adding to these theories that emphasize social identity and the stress of social rejection concerns, the internalization of weight stigma^77^ may play an independent role in explaining why self‐identification is associated with ill health.

Common to many theoretical accounts of the damaging effects of stigma is the consideration that the experience of discrimination due to body weight is common among people living with obesity and experiencing discrimination because of one's weight is predictive of a range of negative health outcomes.78,79. Although self‐identifying as having overweight may make a person more likely to identify experiences of discrimination due to body weight, the outlined model suggests that the damaging effects of self‐identifying as having overweight occur irrespective of whether or not a person has experienced discrimination due to body weight. Although large numbers of people do not identify that they have overweight, self‐identification of overweight among individuals who are medically defined as being overweight (BMI 25–29.9 kg/m2) is still relatively common, for example, 50% of people or more80 and as discussed in this review, self‐identification of overweight among people in this weight range is reliably associated with worse health outcomes. Population‐level studies suggest that only a small minority of people (typically less than 10%) who are medically defined as being overweight report having ever been the victim of discrimination because of body weight.81,82 Therefore, in this group of people, self‐identification of overweight is likely to lead to weight gain and impaired mental health even if a person has never experienced personal discrimination due to body weight.83 Personal experiences of discrimination due to body weight are far more common among people currently living with obesity.81,84 Because of this, personally experiencing discrimination due to body weight may therefore primarily act to exacerbate obesity and obesity‐related ill health, rather than explain the initial development of obesity.

## 9 FUTURE DIRECTIONS

In discussing the evidence linking self‐identification of overweight to health behaviours and outcomes, this article focused on areas that have been studied in sufficient detail to produce convincing evidence (i.e., systematic reviews of the associations between self‐identification and weight control behaviours, depression, and suicidality). However, in individual studies, self‐identification of overweight has been linked to other health relevant outcomes, such as substance misuse and other “risky behaviours”.^85^ Therefore, further research examining a broader range of health outcomes associated with self‐identification of overweight would now be informative.

The proposed model focuses on key psychological and behavioural pathways that link self‐identification of overweight to mental and physical health outcomes that we believe currently has the most convincing evidence. There will, however, invariably be other mechanisms and pathways which will be important to consider in the future. As has been recently suggested,^48^, ^49^ the stigma associated with self‐identifying as having overweight in a society that derogates heavier body weight may cause a chronic stress response to this social‐evaluative threat that has direct physiological effects (e.g., hypothalamic‐pituitary‐adrenal axis activation affecting appetite and body fat deposition), as opposed to impairing health only through lifestyle behaviours. See Wu and Berry^86^ for a systematic review on weight stigma and physiological health outcomes. Also outlined is the proposal that social rejection concerns and stigma internalization are psychologically distressing and primarily it is this distress that makes effortful self‐regulation of behaviour more difficult. However, it is plausible that social rejection concerns and/or stigma internalization may impact behaviour through other channels. Social rejection concerns may also result in some people behaving in less healthy ways (e.g., reduced physical activity), not because of experiencing distress directly, but because people choose to avoid potentially distressing situations in which they will be judged negatively (e.g., exercising in public).^87^ Likewise, the proposed model will likely need to be updated as evidence emerges pointing to novel psychological processes that connect self‐identification to impaired health. For example, because body dissatisfaction is common among young people of heavier body weight^88^ and a risk factor for disordered eating in adolescence,^89^ it may play a role in explaining why self‐identification of having overweight results in unhealthy dieting behaviours and subsequent weight gain, although this possibility is yet to be directly tested.

Although there is evidence for each of the pathways outlined in the proposed model, understanding of weight perception and health will invariably benefit from more stringent and direct testing. The temporal order proposed (e.g., self‐perception of overweight increases likelihood of social rejection concerns and weight bias internalization) is intuitive, but has not been prospectively tested. Furthermore, a large amount of the evidence generated to date is observational in nature. Whilst it can be concluded from this research that people who self‐identify as having overweight go on to experience worse health outcomes in the future than those who do not, experimental work is needed to determine causality and explain why self‐identification of overweight leads to worse health outcomes. Likewise, the suggestion that self‐identification of overweight may be an important psychological process in the progression from overweight to obesity and then the maintenance of obesity would benefit from direct testing. In line with the observation that a minority of people can lose weight and then maintain that weight loss in the long term,^90^ there will also be some people who gain weight, self‐identify as having overweight, and then lose that weight and return to a normal body weight, as opposed to experiencing the pathways outlined in the model.

Demographic and contextual factors may act to modify the health effects of self‐identifying as having overweight. Demographic factors, such as gender and ethnicity, determine the likelihood that a person self‐identifies that they are overweight (e.g., perception of overweight is more common in females than males).^14^ Although this will predispose women to be more likely to subsequently experience the effects of self‐identifying as being overweight, evidence to date does not consistently suggest that the size of association between self‐identification of overweight and weight‐related or mental health outcomes differs by gender.^35^, ^40^ Likewise, although body size will determine the likelihood that a person identifies that they have overweight, the associations between self‐identifying as being overweight and poorer weight management and mental health appear to be observed consistently irrespective of actual body size.^35^, ^40^ For example, both people who are overweight who correctly identify that they are overweight and people with normal weight who incorrectly believe they have overweight exhibit worse weight management and mental health than do people of a similar body weight who do not identify as having overweight.^35^, ^40^ To our knowledge, no research has examined whether there are psychological individual differences that may exacerbate or minimize the damaging effects of self‐identified overweight, but there is a wealth of personality literature on obesity. For example, neuroticism is a predictor of poorer weight management,^91^ and the emotional instability associated with high neuroticism may exacerbate the perceived stigma of self‐identifying as having overweight. Understanding whether some people can effectively manage the stigma attached to being overweight and not experience the negative consequences of self‐identification of overweight would now be informative.

Contextual factors may also have relevance. Ethnic and/or cultural differences in the stigma attached to heavier body weight may result in self‐identification of overweight being more or less damaging. For example, although obesity is prevalent in the United States and this may have subtly changed body size preferences,^92^ the increased prevalence of obesity in the United States has resulted in stigmatizing public health messages about obesity, the expansion of a multimillion‐dollar dieting industry, and media portrayals that reinforce the message that heavier body weight is a personal characteristic to be avoided at all costs.^93^ Although heavier body weight tends to be a widely stigmatized characteristic, levels of weight stigma are thought to differ across countries,^94^ and this will have relevance to the consequences of self‐identification of having overweight. Central to stereotype threat accounts of stigma is the proposition that people experience negative psychological outcomes when exposed to situations or contexts in which their stigmatized identity and/or the associated negative stereotypes are made salient.^95^, ^96^ The extent to which self‐identification of overweight leads to negative psychological outcomes is likely to be in part determined by how frequently people who self‐identify as overweight are exposed to stigmatizing messages or images about heavier body weight. Likewise, other demographic and socio‐cultural factors (race/ethnicity, gender, social class) may be of relevance, and research exploring this may be informative.

## 10 IMPLICATIONS

The finding that self‐identification of overweight is associated with worse mental and physical health outcomes (including weight gain) has public health implications for intervention efforts against overweight and obesity. The long‐held belief that large numbers of people living with overweight and obesity to accurately identify their weight status will be detrimental to their long‐term health now needs revision.^22^ Public health interventions that provide body weight “feedback” are common. The use of public health interventions that provide feedback on child or adolescent weight has been a contentious issue because of concerns over potential adverse effects on body image and mental health.^97^ Based on the research reviewed, providing feedback on weight status alone is unlikely to be an effective way of preventing further weight gain or promoting weight loss. To date, the effectiveness of “weight feedback” interventions has received limited testing^23^ and therefore one concern is that if intervention approaches that draw attention to body weight do not also address the stigma of self‐identifying as having overweight, they may inadvertently promote rather than prevent or reduce overweight and obesity.^46^

Moving beyond intervention approaches that provide weight feedback, the findings discussed here support calls for overweight and obesity public health interventions to more carefully consider the stigma associated with heavier body weight and to ensure that they do not serve to further stigmatise overweight and obesity. One way of achieving this is by public health messages placing less emphasis on body weight and instead place a greater emphasis on the importance of behaviours that are both health‐protective and support weight management. Furthermore, by directing messages about the importance of health‐protective behaviours to all (irrespective of weight status), this itself is likely to be less stigmatizing and addresses the need for prevention of overweight and obesity. More importantly, public health approaches that change the environment to make healthier eating and physical activity easier or default choices will likely benefit population‐level health without overt stigmatization.^39^ The research discussed may also have relevance to people actively attempting weight loss. Long‐term weight loss is notoriously difficult to achieve,^61^ and one reason may be the stigma and related psychological antecedents of self‐identifying as being an overweight person. Approaches that attempt to reduce or protect against one or more of the key psychological processes outlined in the proposed model (e.g., by reducing weight bias internalization) may therefore improve long‐term weight loss outcomes and allow for empirical testing of the model proposed. For example, cognitive reappraisal strategies may reduce internalization of weight bias and in doing so reduce maladaptive coping responses caused by internalization of weight bias.

The extent to which the research discussed on self‐identification of being overweight, stigma, and health outcomes is relevant to understanding of why heavier body weight is associated with increased risk of ill health and early mortality also warrants consideration. Plausible pathways through which the stigma associated with identifying as having overweight may explain part of the obesity‐related disease burden have been discussed. For example, the observation that it is self‐identification of overweight (rather than being overweight per se) that predisposes a person to increased risk of developing depression^40^ is consistent with the proposition that the psychological experience of “being an overweight person” may be more damaging to mental health than actually being overweight. Moving beyond the effects of self‐identifying as having overweight, recent research speaks more directly to this suggestion: the tendency for objectively measured obesity to be associated with worsening of physiological health (e.g., biomarkers of underlying cardiovascular and metabolic health) is an expected finding from the biomedical literature. However, in a cohort of older English adults, this link between obesity and worsening physiological health was largely explained by the amount of stigma that participants with obesity reported experiencing^98^: experiencing discrimination due to body weight predicted greater physiological dysregulation over time and after accounting for the weight discrimination experienced by participants with obesity, the association between objectively measured obesity and worsening physiological health was no longer statistically significant. In line with recent calls,^99^ evidence suggests that weight stigma may be an important reason why excess body weight places people at increased risk of developing ill health.

## 11 CONCLUSIONS

People who identify that they have overweight are more likely to experience worsening mental and physical health than those who do not identify that they have overweight. The widespread stigma associated with heavier body weight may explain this counter‐intuitive finding: the psychosocial experience of “being overweight” may result in a myriad of psychological effects that increase the likelihood of engaging in unhealthy lifestyle behaviours and suffering ill health. In the context of overweight and obesity, knowing may hurt and ignorance may be bliss.

## CONFLICTS OF INTEREST

All authors report no conflicts of interest. ER has received research funding from the American Beverage Association and Unilever for unrelated research.
